# Three-Dimensional Virtual and Printed Prototypes in Complex Congenital and Pediatric Cardiac Surgery—A Multidisciplinary Team-Learning Experience

**DOI:** 10.3390/biom11111703

**Published:** 2021-11-16

**Authors:** Laszlo Kiraly, Nishant C. Shah, Osama Abdullah, Oraib Al-Ketan, Reza Rowshan

**Affiliations:** 1 Division of Pediatric Cardiac Surgery, Cardiac Sciences, Sheikh Khalifa Medical City, Abu Dhabi P.O. Box 51900, United Arab Emirates; 2 Department of Public Health, Semmelweis University, H-1085 Budapest, Hungary; 3 Department of Cardiac, Thoracic and Vascular Surgery, National University Hospital System, 1E Kent Ridge Road, NUHS Tower Block, Level 9, Singapore 119228, Singapore; 4 Department of Surgery, Yong Loo Lin School of Medicine, National University of Singapore, 1E Kent Ridge Road, NUHS Tower Block, Level 8, Singapore 119228, Singapore; 5 Division of Pediatric Cardiology, Cardiac Sciences, Sheikh Khalifa Medical City, Abu Dhabi P.O. Box 51900, United Arab Emirates; nishah@seha.ae; 6 Core Technology Platform Operations, New York University Abu Dhabi, Abu Dhabi P.O. Box 129188, United Arab Emirates; oa22@nyu.edu (O.A.); oga2@nyu.edu (O.A.-K.); reza.rowshan@nyu.edu (R.R.)

**Keywords:** three-dimensional printing, congenital heart disease, congenital heart surgery, surgical simulation, surgical training, hands-on surgical training

## Abstract

Three-dimensional (3D) virtual modeling and printing advances individualized medicine and surgery. In congenital cardiac surgery, 3D virtual models and printed prototypes offer advantages of better understanding of complex anatomy, hands-on preoperative surgical planning and emulation, and improved communication within the multidisciplinary team and to patients. We report our single center team-learning experience about the realization and validation of possible clinical benefits of 3D-printed models in surgical planning of complex congenital cardiac surgery. CT-angiography raw data were segmented into 3D-virtual models of the heart-great vessels. Prototypes were 3D-printed as rigid “blood-volume” and flexible “hollow”. The accuracy of the models was evaluated intraoperatively. Production steps were realized in the framework of a clinical/research partnership. We produced 3D prototypes of the heart-great vessels for 15 case scenarios (nine males, median age: 11 months) undergoing complex intracardiac repairs. Parity between 3D models and intraoperative structures was within 1 mm range. Models refined diagnostics in 13/15, provided new anatomic information in 9/15. As a team-learning experience, all complex staged redo-operations (13/15; Aristotle-score mean: 10.64 ± 1.95) were rehearsed on the 3D models preoperatively. 3D-printed prototypes significantly contributed to an improved/alternative operative plan on the surgical approach, modification of intracardiac repair in 13/15. No operative morbidity/mortality occurred. Our clinical/research partnership provided coverage for the extra time/labor and material/machinery not financed by insurance. 3D-printed models provided a team-learning experience and contributed to the safety of complex congenital cardiac surgeries. A clinical/research partnership may open avenues for bioprinting of patient-specific implants.

## 1. Introduction

“The essence of the virtual world is the freedom it allows for experimentation” [1].

Anatomical modeling of the patients’ individual three-dimensional (3D) structures and 3D printing of the prototypes has won its place in personalized medicine and reconstructive surgery [2]. There are two types of 3D-printed objects in healthcare, as shown in Table 1.

At present, pediatric and congenital cardiac surgery only utilizes ‘type 1′ anatomic models that promote a better understanding of complex anatomy by combining visual and tactile stimuli [4]. They are employed to plan and emulate the operation preoperatively. Models also improve communication within the multidisciplinary clinical team and towards patients and their relatives [5]. 3D models often bring new anatomical information and open the possibility of elaborating [6,7] and testing alternative surgical scenarios [8]. All these options are available before the actual intervention, so increased awareness contributes to greater procedural safety [9,10,11].

In this study, we report our single-center experience with 3D modeling and printed models in surgical planning of complex congenital cardiac surgery as an interdisciplinary team-learning process to realize and validate possible clinical benefits. We also present a sustainable co-operation model between applied/basic science and the clinical practice that broadens the possibilities of a learning organization and can overcome constraints in the financing of the prototypes and may initiate further developments towards the construction of ‘type 2′ 3D-printed patient-specific implants: patches, valves, and conduits.

## 2. Materials and Methods

This study was carried out at Sheikh Khalifa Medical City (SKMC), a government-owned, teaching hospital in the United Arab Emirates. There are approximately 150 advanced cardiac imaging procedures, around 150–200 mostly interventional cardiac catheters, and 400 congenital heart surgeries performed annually at this institution. Pediatric cardiac services offer tertiary care, comprehensive and uninterrupted treatment coverage for the majority of the population (approximately 9 million) [12]. The surgical patient population is skewed towards the complex, younger patients requiring more urgent interventions. Case scenarios requiring 3D virtual modeling and 3D printing were elected from the subgroup of patients requiring reoperations with the most complex anomalies (with Aristotle Basic Complexity Score over 10) [13] (approximately 10% of surgical patient material) spanning over a period of 18 months of the introduction of 3D-printed models. Indication for 3D-printed models was raised at the multidisciplinary meeting for a better understanding of the complex anatomical situation, consideration of alternative surgical solutions, intervention planning, and rehearsing. Institutional Research Ethics Committee review and approval were waived for this study due to the fact that anatomical models classified as research models were not coming into direct contact with the patients. Informed consent was obtained from the patients/guardians. Non-clinical participants of the study strictly adhered to patient data confidentiality.

### 2.1. Financing

In an earlier phase of the project, segmentation and model printing was outsourced to Materialise, Leuven, Belgium, and expenses were covered by a grant from Hamdan bin Rashid Al Maktoum Foundation for Distinguished Academic Performance, Dubai, UAE. In the second phase, a cost-efficient sustainable manufacturing and financing model was established in a clinical/research partnership with the Core Technology Platforms at New York University Abu Dhabi (NYUAD).

### 2.2. Imaging and Manufacturing

**Figure 1:**
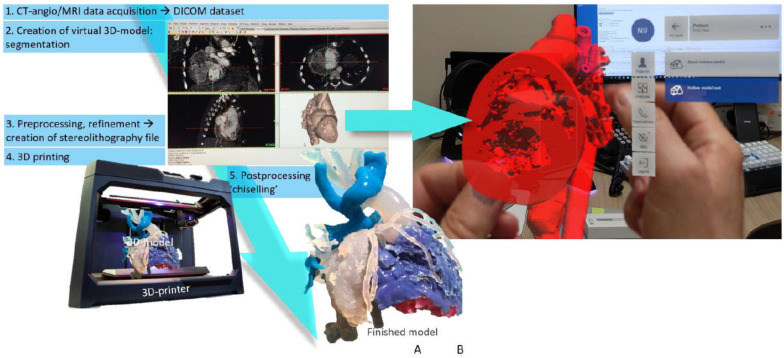
Production phases of 3D modeling for printed prototypes and holograms. (**A**) Digital raw data acquired from imaging sources (CT angiography, MRI) undergo segmentation to create a 3D virtual model. After preprocessing refinement, the stereolithography file is 3D-printed. Models receive postprocessing treatment. (**B**) 3D virtual model as a hologram can also be imported into virtual reality, where it can be fully rotated and entered.

ECG-gated computed tomography (CT) was performed combined with contrast-enhanced angiography recorded during expiration at a resolution of 0.3 to 0.7 mm. CT was done within 6 weeks prior to the planned surgical procedure. A virtual model (segmentation) was created from the digital imaging and communication in medicine (DICOM) data set using commercial software (Mimics^®^, Materialise, Leuven, Belgium [14]) and an open-source 3D Slicer [15]. In all cases, a research engineer carried out semi-automatic segmentation with the clinician’s participation. The segmentation steps involved loading the DICOM data into Mimics or 3D Slicer, using a combination of intensity-based thresholding to segment the blood pool signal, and creating a hollow model of 2–3 mm wall thickness to outline the surface of the vessels. Manual segmentation of the heart tissue was performed when needed for anatomical clarity. Manual model refinement (preprocessing) was done at the end and approved by clinicians. A fully-rotatable and sliceable virtual 3D model of the cardiac structures was presented in a pdf file on the computer’s screen (2D). From the stereolithography (STL) file, a life-size ‘blood volume’ model made of VeroMagenta (Stratasys, Eden Prairie, MN, USA), hard opaque material and another 1.5× scaled ‘hollow’ made of TangoPlus (Stratasys, Eden Prairie, MN, USA) and/or HeartPrint Flex (Materialise, Leuven, Belgium), a flexible, translucent material, models were printed (see below). Magnification of the hollow models was used to facilitate surgical emulation. Figure 1 illustrates the steps of 3D modeling and creating 3D-printed prototypes and holograms.

The printed prototypes were classified as ‘research models’ and did not come into direct contact with the patient’s tissues [16]. The blood volume model was utilized for exact measurements of anatomical structures, such as vessel diameters. The hollow model featured endo- and epicardial surfaces, i.e., the wall thickness of the given heart cavity and/or great vessel between them. In order to simulate natural tissues, material characteristics of the hollow model were chosen to be similar to that of the human arteries (Young’s modulus between 0.2 and 9 MPa; distensibility between 1.2 × 10^−3^ and 6.6 × 10^−3^ mmHg^−1^) and, to practice surgical emulation enlarged models (scale 1.5×) were created. The accuracy of the 3D-printed models was cross-checked with the intraoperative surgical conditions.

### 2.3. Manufacturing of the 3D-Printed Models

3D printing was performed using the Stratasys J750 resin-based 3D printer (Stratasys, Eden Prairie, MN, USA). This multijet 3D printing technology allows concurrent jetting and UV-curing of several materials. The materials have different physical properties and can be mixed to obtain a range of intermediate physical properties and colors that would match the properties and colors of the different tissues. The digital 3D models obtained from the image segmentation were sliced and 3D-printed layer by layer. The empty domains within the model were filled with water-soluble support material. After 3D printing, the models were submerged in a water bath with 2% NaOH and 1% Na_2_SiO_3_ to remove the support.

### 2.4. Preoperative Team Emulations

Repeated rehearsals and emulation sessions were held with the intraoperative team as well as with the multidisciplinary team. The added value and acceptance of models in clinical decision making were assessed as a team-learning experience. Members in the multidisciplinary team and the relatives of patients gave informal feedback and filled questionnaires in throughout the project. Questionnaires polled the opinion separately for the medical and non-medical participants on the added value of 3D-printed models over virtual ones, model accuracy and cost/benefit adequacy, and medical and non-medical participants’ willingness to assume extra work and expenses related to model production.

## 3. Results

### 3.1. Patient Characteristics and Material

3D virtual and printed models of the heart and the great arteries were created for fifteen complex congenital cardiac patients (nine boys, six girls, median age: 11 months; range: 6.5–96 months) in preparation for cardiac surgery. Thirteen out of fifteen patient (86.66%) were planned for redo-operations, in 10 cases as part of uni- or biventricular staging. No operative morbidity/mortality occurred. Table 2 shows the study characteristics of the patients.

**Figure 2:**
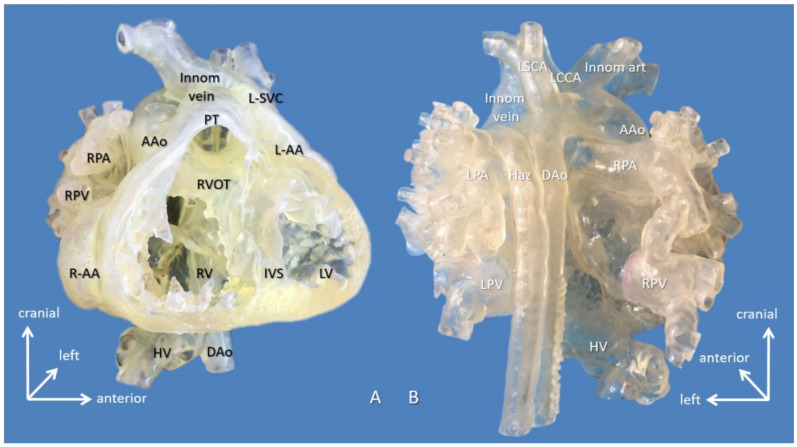
3D-printed hollow model of right atrial isomerism, visceral heterotaxy, dextrocardia, common atrium, and hemiazygos continuity to left superior vena cava (Case 11). (**A**) Right anterior oblique view with the ventricular apex removed. Innominate vein and the interrupted inferior vena cava connected by the hemiayzgos vein drain into left-sided atrium via left superior vena cava. The hepatic veins also drain to the left-sided atrium. (**B**) Posterior view. Abbreviations: AAo: ascending aorta, DAo: descending aorta, Haz: hemiazygos vein, HV: hepatic vein, innom art/vein: innominate artery and vein, IVS: interventricular septum, L-AA: left-sided morphologically right atrial appendage, LCCA: left common carotid artery, LPA: left pulmonary artery, LPV: left pulmonary vein, LSCA: left subclavian artery, L-SVC: left superior vena cava, LV: left ventricle, PT: pulmonary trunk, R-AA: right-sided morphologically right atrial appendage, RPA: right pulmonary artery, RPV: right pulmonary vein, RV: right ventricle.

Anatomical complexity of the patients was characterized by the high prevalence of positional anomalies (dextrocardia/mesocardia: 8/15 = 53.33%), visceral heterotaxy (5/15 = 33.33%), and anomalies of the systemic or pulmonary venous return (7/15 = 46.66%). Figure 2 demonstrates a case scenario (Case 11) with right atrial isomerism and hemiazygos continuity of the interrupted inferior vena cava planned for interatrial baffle completion.

**Figure 3:**
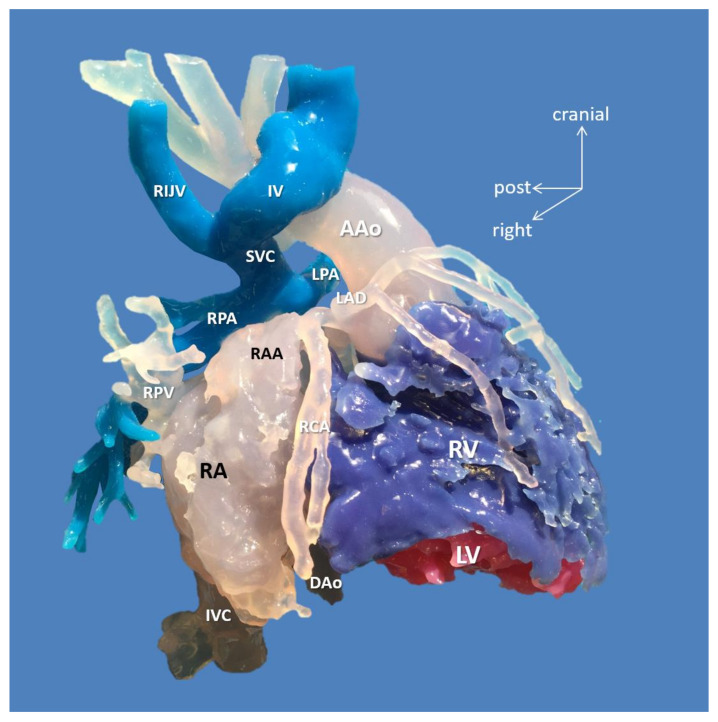
3D-printed blood volume model of mesocardia, common atrium, criss-cross heart (supero-inferior ventricles), transposition of the great arteries, and pulmonary atresia, restrictive VSD and thrombus formation in the left ventricle; operated bidirectional superior cavopulmonary (Glenn) anastomosis (Case 15). Patient also had variant coronary artery anatomy: right coronary and left anterior descending arteries originated from left-hand facing posterior sinus, and a separate circumflex originated from right-hand facing anterior sinus. Modeling was indicated to assess the extent of the left ventricle thrombus and suitability for biventricular repair. The model did not reveal any possibility of connecting the left ventricle to the aorta. Patient underwent univentricular staging: total cavopulmonary connection with intracardiac conduit, LV thrombus removal and VSD enlargement. Abbreviations: AAo: ascending aorta, DAo: descending aorta, IV: innominate vein, IVC: inferior vena cava, LAD: left anterior descending coronary artery, LPA: left pulmonary artery, LV: left ventricle, RA: right atrium, RAA: right atrial appendage, RCA: right coronary artery, RIJV: right internal jugular vein, RPA: right pulmonary artery, RPV: right pulmonary vein, RV: right ventricle, SVC: superior vena cava.

Despite the complex anatomy, restoration of biventricular circulation was possible for patients with two ventricles (10/15), but in one scenario (Case 15, Figure 3), both the 3D virtual and printed models were extremely helpful in disproving the feasibility of reconnection of the left ventricle to the aorta, and thus, biventricular circulation.

**Figure 4:**
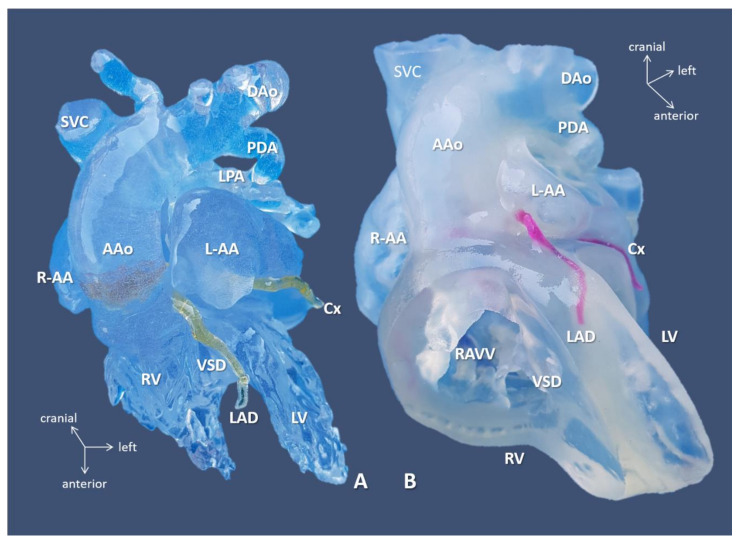
3D-printed blood volume (**A**) and hollow (**B**) models of right atrial isomerism, visceral heterotaxy, and dextrocardia (Case 10). Anterior view: free wall of the ventricles is removed on the hollow model. Complex anomalies comprised of left-sided IVC; right-sided SVC receives inflow from common pulmonary vein, i.e., supracardiac total anomalous pulmonary venous return (see, below); common atrium, complete AV defect; double outlet right ventricle/transposition of the great arteries with pulmonary atresia. 3D-printed models were instrumental in planning for complete biventricular repair the patient successfully underwent subsequently. Abbreviations: AAo: ascending aorta, Cx: circumflex coronary artery, DAo: descending aorta, L-AA: left-sided morphologically right atrial appendage, LPA: left pulmonary artery, LV: left ventricle, PDA: patent arterial duct, R-AA: right-sided morphologically right atrial appendage, RAVV: right AV valve, RV: right ventricle, SVC: right-sided superior vena cava, VSD: ventricular septal defect.

**Figure 5:**
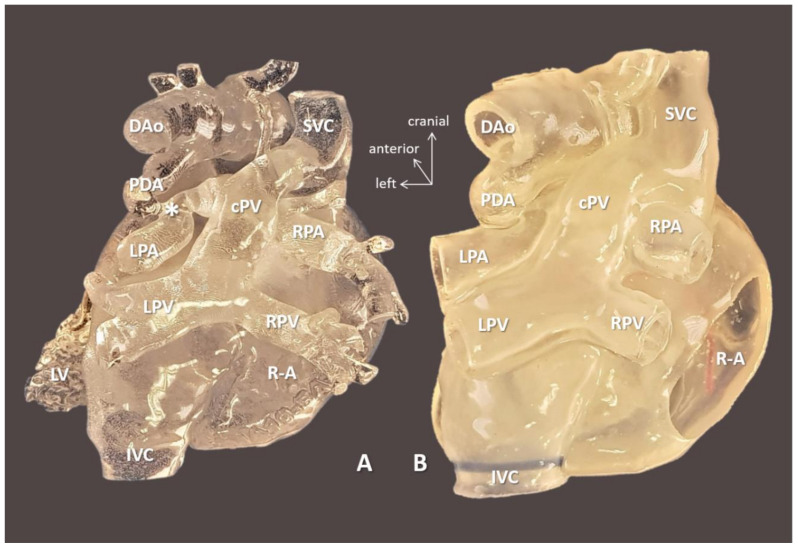
3D-printed blood volume (**A**) and hollow (**B**) models of right atrial isomerism, visceral heterotaxy, and dextrocardia (Case 10). Posterior view: right-sided atrium is opened on the hollow model. Complex anomalies are illustrated on the models left-sided IVC; right-sided SVC receives inflow from common pulmonary vein (cPV), i.e., supracardiac total anomalous pulmonary venous return. Tortuous patent arterial duct (PDA) reaches the left pulmonary artery (LPA); there is pulmonary coarctation (*) at the entry point. The models were instrumental in planning for complete biventricular repair the patient successfully underwent subsequently. Abbreviations: cPV: common vertical pulmonary vein, DAo: descending aorta, IVC: left-sided inferior vena cava, LPA: left pulmonary artery, LPV: left pulmonary vein, LV: left ventricle, PDA: patent arterial duct, R-A: right-sided atrium, RPA: right pulmonary artery, RPV: right pulmonary vein, SVC: right-sided superior vena cava.

Biventricular repairs (9/15 = 60%)—mostly (re)operations—associated with an Aristotle Basic Complexity Score [13] of the mean of 10.64 ± 1.95. Owing to detailed and strategic surgical rehearsing on the 3D models, successful complete biventricular repair—consisting of repair of pulmonary venous stenosis, atrial separation, AV-valve repair, intraventricular rerouting, take-down of previous superior bidirectional cavopulmonary anastomosis, and implantation of RV-PA conduit—could be performed for the most complex case scenario (Case 10) demonstrated on Figure 4 and Figure 5.

**Figure 6:**
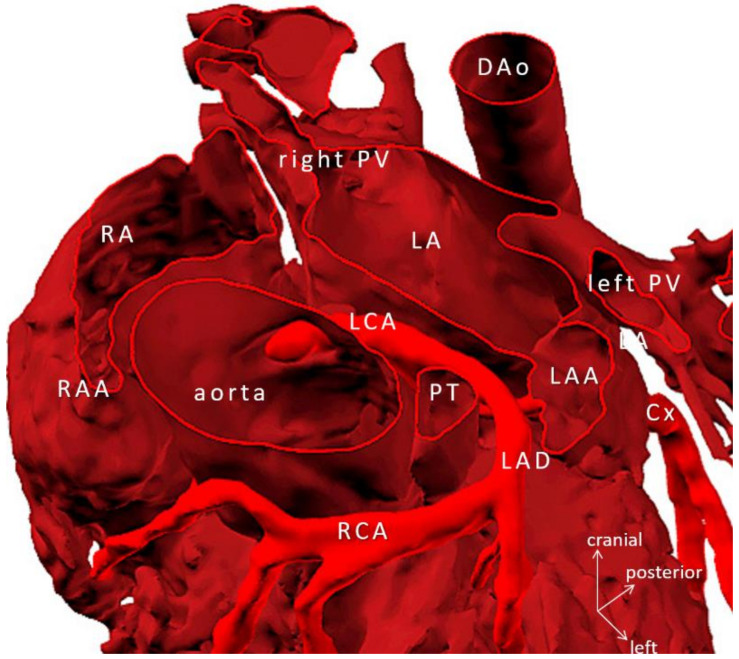
Virtual 3D model tetralogy of Fallot, pulmonary atresia with anomalous origin of the right coronary artery from left anterior descending branch of the left coronary artery (Case 5). Ascending aorta is transected at the level of the sinotubular junction. Note: knowledge of the exact course of the aberrant coronary artery is crucial in avoiding injury during the placement of the right ventricle to pulmonary bifurcation conduit. Abbreviations: Cx: circumflex branch of the left coronary artery, DAo: descending aorta, LA: left atrium, LAA: left atrial appendage, LCA: mainstem left coronary artery, LAD: left anterior descending coronary artery, PT: pulmonary trunk, PV: pulmonary vein, RA: right atrium, RAA: right atrial appendage, RCA: right coronary artery.

Patients with univentricular physiology (6/15 = 40%) could have progressed on their staging, except for one patient with intractable pulmonary hypertension (Case 4). Three patients (20%) had significant issues with coronary artery origin and/or course anticipated from clinical imaging but only fully revealed on the virtual and/or printed 3D model. Figure 6 shows the anomalous origin of the right coronary artery from the left anterior descending branch of the single left coronary artery. Operative procedures were performed along with preoperative plans; no deviation from the rehearsed steps occurred. No reoperation during follow-up was necessary.

### 3.2. Indication for 3D Modeling and Printing

Indication of 3D modeling was (1) to further illuminate the spatial relationship in the segmental anatomy and for 3D printing (2) to facilitate surgical planning. 3D virtual models were always available, from which blood volume (N = 10) and hollow (N = 9) prototypes were printed. In three cases, analysis of the virtual 3D model was sufficient to refine the anatomy and formulate a surgical plan. These scenarios revealed (1) abnormal right coronary artery from the left anterior descending branch (Case 5), (2) an obstructed left main coronary orifice compressed by a dilated right pulmonary artery (Case 6), and (3) exact location of left-sided intra-atrial obstruction (Case 8).

### 3.3. Added-Value and Accuracy of 3D Modeling/Printing

Exact size measurements were taken from blood volume models. The shapes of implants in relation to the intracardiac defects were assessed, and steps of the repair were designed and rehearsed on the hollow models. Registration of the AV valves (on the CT and/or MRI dataset) is currently suboptimal to include them in the 3D-printed models.

**Figure 7:**
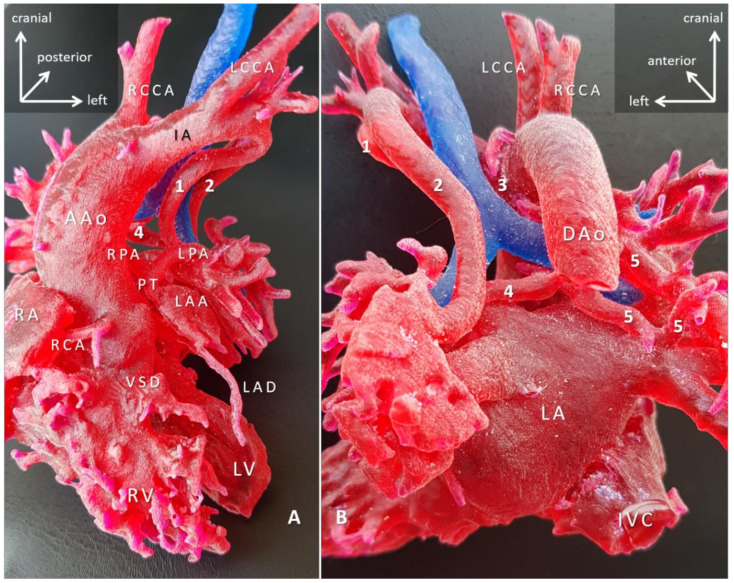
3D-printed blood-volume model of pulmonary atresia, VSD and major aortopulmonary collaterals arteries (MAPCAs); right aortic arch (Case 7). Blood-volume model printed in red, and trachea printed in blue to give anatomical reference. (**A**) Anterior view; (**B**) posterior view. The right ventricle outflow tract is missing, and the native pulmonary arteries are hypoplastic. The pulmonary circulation entirely depends on the MAPCAs (1–5). The surgical task is paramount that involves reconstruction of the intrapericardial pulmonary arteries by unifocalization of all five MAPCAs and connecting them to the right ventricle via a preferably valved and growing conduit, with/out closure of the VSD. The surgical plan was worked out in detail with the 3D-printed model. Abbreviations: AAo: ascending aorta; DAo: descending aorta; IA: innominate artery; IVC: inferior vena cava; LA: left atrium; LAA: left atrial appendage; LAD: left anterior descending branch of the left coronary artery; LCCA: left common carotid artery; LPA: left pulmonary artery; LV: left ventricle; PT: pulmonary trunk; RA: right atrium; RCA: right coronary artery; RCCA: right common carotid artery; RPA: right pulmonary artery; RV: right ventricle; VSD: ventricular septal defect.

Virtual and printed 3D models revealed new anatomic findings relevant for the surgery in 9/15 (60%). For three patients (Cases 3: site of subaortic resection; Case 6: external coronary compression as the cause of LV failure; and Case 15: the presence of a VSD obstructed by LV thrombus) previously classified inoperable due to high surgical risk, 3D models gave new insights in the anatomy and opened the possibility of setting up an alternative surgical plan. In other words, these patients became ‘operable’. Models also had a high yield in clarifying the surgical anatomy (13/15 = 86.66%) that translated into specific recommendations on the surgical approach (e.g., which cardiac chamber should be opened), perfusion technique (e.g., where to cannulate), surgical steps of complex repairs. The added value of the 3D-printed models is that they enabled surgical emulation and preprocedural planning for the shape and size patches, conduits needed [17]. Figure 7 demonstrates precise anatomic details of complex pulmonary atresia with MAPCAs (Case 7). Hands-on use of the 3D-printed blood-volume model was essential to assess the spatial relationship of the MAPCAs and the tracheobronchial tree and to rehearse the plan of unifocalization. For the lack of an adequate control group, we could not draw comparisons on the 3D models’ impact on saving surgical time. Intraoperative identification of the structures seen on the models, however, reportedly created a ‘déjà vu’ effect, and it improved the surgical flow and confidence in executing the complex operations.

**Figure 8:**
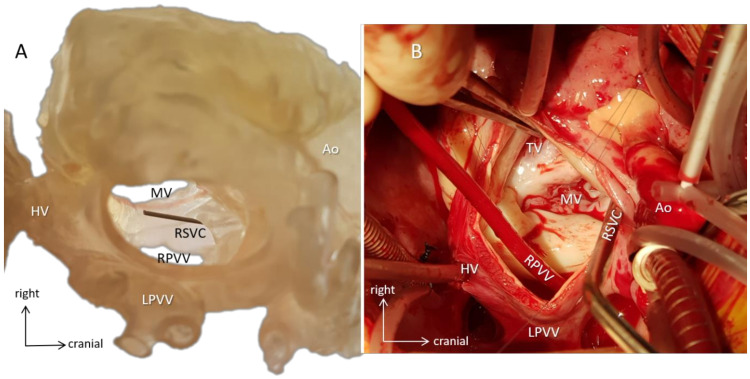
Surgical simulation. Case 13 with dextrocardia (mirror-image arrangement, bilateral SVCs, hemiazygos continuity of interrupted IVC, common atrium, incomplete AV defect, valvar pulmonary stenosis, vascular ring). (**A**) A 3D-printed hollow model viewed from the orientation of the surgeon standing on the left side of the patient demonstrates the intracardiac anatomy. A probe emerges in the mouth of the right superior vena cava (RSVC). By identifying anatomical landmarks, e.g., the AV valves and the entrances of the pulmonary and hepatic veins, surgical steps can be simulated, and size and shape of the baffle can be designed preoperatively. (**B**) Intraoperative representation of the same anatomy. The surgeon identifies structures already familiar with from the 3D model (e.g., metal suction tube is in the right superior vena cava), and the course of the operation progresses along with the preoperative plans. The 3D model and the intraoperative image are closely matched. Abbreviations: Ao: aorta, HV: hepatic veins, LPVV: left-sided pulmonary veins, MV: mitral valve, RPVV: right-sided pulmonary veins, RSVC: right superior vena cava, TV: tricuspid valve.

Accuracy of the 3D virtual models showed exact matching with the CT-dataset they were based on. Accuracy depended on two factors, manual segmentation for the virtual model and 3D printed resolution for the printed model, which was always better than imaging resolution. 3D-printed models were then validated by comparing the diameters of the inferior vena cava and ascending aorta from the CT scan and comparing with those from the blood volume 3D-printed model (data were extrapolated for the scaled prototypes). No significant difference was found between CT scans, the virtual, and 3D-printed models. The 3D-printed prototypes were also evaluated with the intraoperative findings, and the models were precise to the 1 mm range (quantitative identifier). No morphological mismatch (qualitative identifier) was found between the 3D-printed models and the intraoperative anatomy in the series. Figure 8 (Case 13) demonstrates the accuracy of the model compared to the intraoperative situation.

### 3.4. Technical, Organizational and Financial Aspects

Creation and printing 3D patient-specific prototypes in this series involved data acquisition (from CT angiography) and segmentation by 2–4 h of computer work with special software and expertise. The printing process required additional 4–6 h depending on the model’s size and complexity. In the starting phase of the project, for the lack of established hardware and infrastructure, outsourcing the production phases to an internationally renowned company (Materialise, Leuven, Belgium) seemed a viable solution. Foundation of clinical/research co-operation with specialized technical expertise for image segmentation and additive manufacturing at NYUAD opened up an avenue for organic development in regular 3D printing and set the basis for related future projects, e.g., in 3D bioprinting. The shift from outsourcing to local co-operation resulted in significant improvements in the speed of manufacturing the prototypes by taking away geographical barriers and provided a sustainable financing structure.

### 3.5. Team-Learning Experience

3D modeling and printing added a new modality for preoperative planning and communication. Sporadic need for 3D models (N = 3) in the first half of the study period of 18 months swapped to a surge of case scenarios (N = 12) in the second half. Rapid learning of the complex cardiac anatomy was observed among clinical engineers supported by clinicians’ participation. Team rehearsals significantly contributed to establishing a shared mental model and stepwise operative plan with alternative scenarios and respective responsibilities among operative team members. Questionnaires on different aspects (Table 3) revealed that virtual 3D models had already provided adequate information for most members of the multidisciplinary team; however, surgeons and patient relatives preferred the hand-held, 3D-printed prototypes. Accuracy was considered as good, and most responders reported improved communication as a primary advantage. Patient relatives were dedicated to subscribing additional costs related to 3D modeling and printing.

## 4. Discussion

This study represents our multidisciplinary team’s learning experience with 3D virtual and printed models in preparing for complex, mostly redo pediatric cardiac procedures. High anatomical and procedural complexity in our series warranted a 3D understanding of the scenarios. 3D-printed models naturally contributed to an interactive team experience; at rehearsals, they allowed that the entire clinical team would appropriate a shared mental image and detailed plan. Parents not familiar with reading images of traditional medical imaging themselves preferred touchable physical objects to virtual ones. Furthermore, interaction with clinical engineers, experts in additive manufacturing, and bioengineers promoted knowledge of each other’s fields that could inspire continuing crosstalk and co-operation in biofabrication.

### 4.1. 3D-Printed Models vs. Modern Imaging Modalities

3D modeling and printing has earned acceptance in congenital cardiac surgery for preoperative decision making, rehearsing, and safe execution of complex procedures [18,19,20,21,22,23]. The risk of open-heart surgery for complex congenital heart defects and in reoperations is still significantly higher compared to other surgical activities prompting for safe surgery measures [24]. Operative efficiency and learning curve are nowadays not expected to impact outcomes [25]. 3D-printed models improve understanding of 3D anatomy and allow anticipation and communication of technical challenges [19,21,24]. Anatomical specimens have a long history, and they significantly contributed to abstracting individual features into general rules, connecting function to structure [26,27,28]. Generations of students of congenital heart disease familiarized themselves with the complex anatomical relationships in the pathological museum [29,30]. The unique advantage of 3D models is that they convey haptic information and binocular vision to complement and strengthen multisensory convergence in creating a mental model of an object [31,32,33]. The strength of palpation is illustrated by that tactile information can even suppress image perception transmitted from the dominant eye under experimental conditions, when the two eyes look at different sights [34,35]. The combination of vision and haptics leads to better analysis, faster decisions, and reduced number of touches [36]. In contrast, advanced clinical imaging, e.g., 3D/4D echocardiography, 3D rotational angiography, CT angiography, and magnetic resonance imaging/MRI, creates 3D models that still exist in the two-dimensional space of a computer screen [36,37]. In order to overcome this possible problem, new technologies have emerged, where the virtual 3D model is projected the virtual and/or mixed reality space [38,39] and the spectator wearing special glasses can interact with them with/out haptic feedback [40,41,42] (Figure 1B). These developing techniques are in the process of maturation and in finding their niche in the clinical armamentarium [43,44,45,46]; however, the need for 3D-printed models remains, as they readily exist in the physical reality and they accurately demonstrate rather complex morphologies [47], which can be printed with the physical properties of native tissues [48].

### 4.2. 3D-Printed Models Promote Team Learning

Planning for complex congenital cardiac operations is both a cognitive process for the operating surgeon [49,50,51] and for the interdisciplinary team [52]. The objective is to build shared mental models of the anatomy, steps of the operation with respective responsibilities, and anticipating avenues to overcome possible complications. Team members, however, are bound by their own perspectives, knowledge gaps, and team dynamics. “Marrying teamwork to one’s own ego is quite difficult at times–and now we’re still learning that” [53]. In the present study, pediatric cardiologists were already content with the 3D virtual models, whereas surgeons and the operative team facing the context of increasing complexity still required the added demonstrating value of 3D-printed models. Non-medical personnel (i.e., patient relatives), being unfamiliar with 2D medical imagery, disfavored virtual models. The learning process, i.e., building a mental model, started with the clinicians’ participation in the segmentation process. Overall, we observed an increasing interest in 3D modeling and printed prototypes demonstrated by the escalating incidence of case scenarios during the study period. Preoperative emulation sessions with the 3D-printed models and with multidisciplinary participation team facilitated shared team-learning experience by taking away time constraints typically present in the operating theatre, allowing reversible actions and repetitions and simplifying complexity. Our experience replicates the reports by others [54,55] that building shared mental models and language promotes shared responsibilities, improved teamwork, and communication. The utilization of 3D-printed models remains a central element of the team experience [56].

### 4.3. Cost-Reimbursement Constraints and Technical Limitations

At present, 3D modeling and 3D printing does not have an internationally accepted current procedural terminology (CPT) code [57]. Since remuneration of treatment activities is based on these codes, their absence represents an obstacle with insurance companies and prompts for alternative funding. Costs of 3D medical modeling and printing are funded by insurance in Japan, and negotiations at the American Medical Association are reportedly underway to include them in the reimbursed activities [58]. A questionnaire survey in the present study showed high willingness among patient relatives to subscribe to extra expenses associated with model production. 3D printing of ‘rigid’ blood-volume models is relatively inexpensive due to the available techniques and single inexpensive material (typically polylactic acid, PLA). However, printing a ‘hollow’ model with flexible, vessel/chamber-mimicking material is an expensive and difficult task because it requires advanced printing capabilities only offered by a handful of 3D printers (such as Stratasys J750 multijet printer, Stratasys, Eden Prairie, MN, USA), using special materials made of multiple resins. A clinical/research institutional co-operation may relieve the financial burden for individual case scenarios, and it can also promote co-operation, e.g., in translational research in the treatment of congenital heart disease.

### 4.4. Future Directions

‘Type 1’ 3D-printed cardiac models do not come to direct contact with the patient; however, computer-aided design and 3D bioprinting can now produce individually implantable (‘type 2’) prototypes in many domains of reconstructive surgery [59,60,61]. Congenital and pediatric cardiac surgery mostly deals with reconstructive patient scenarios when defects are closed, various segments of the heart are connected, valves are implanted. Congenital cardiac surgery applies prosthetic material in the majority (>75%) of its procedures [62]. However, biomaterials currently available in our profession lack the potential of growth, the implanted conduits and valves derange over time surrendering recipients to repeated reoperations [63], and currently, numerous projects address biofabrication of the patches, valves or conduits [64,65,66]. 3D-printed prototypes can play a significant role in biofabrication as templates for computer-aided design, bioscaffolds [67]. Availability of patient-specific, autologous (i.e., non-immunogenic), structurally sound, viable and growing implants could cancel reoperations and could entail improved quality-of-life of the individual patient and relief of a significant public health burden [68]. We believe that clinical/research co-operation in biofabrication and 3D bioprinting—as in our study—holds the promise of realizing these future goals [69,70].

As another direction, rapid development in holographic technology carries potentials for 3D modeling. Holograms offer quicker production time and low budget solution compared to 3D printing [45,46]. Applications allow free maneuverability, free magnification, the possibility of virtual tours inside the cardiac chambers and segments as a shared 3D team experience. Superimposed holograms on intraoperative scenarios are currently introduced as surgical decision making and adjuvant tool in several surgical disciplines [71]. Provided that computational strength increases in future versions, higher-resolution cardiac-cycle models could be imported into the virtual/mixed reality from dynamic imaging sources, e.g., 3D echocardiography, structure and function will conjoin [45]. The application will also solve the problem of the currently suboptimal representation of the cardiac valves [45].

### 4.5. Study Limitations

This report is a single-center, initial experience of 3D modeling and printing focusing on complex congenital cardiac surgical scenarios without a control group. Technical, financial and logistic limitations are represented in focusing on the most complex, mostly redo case scenarios; thus, potential selection bias may exist. The advantages of 3D modeling and printing mostly rely on qualitative opinion. No quantitative data are currently available on reduction of complications, savings in operating time. A multicentric study is also warranted to formulate a strong case for financial reimbursement. By the essence of the data acquisition methodology, registration of the cardiac valves is suboptimal, and current 3D models are static images addressing the structure rather than cardiac function.

## 5. Conclusions

3D modeling and 3D printing is a relatively new modality in congenital cardiac surgery based on multi- and interdisciplinary teamwork. It offers multiple advantages in team learning for safe surgery, education, and communication. At present, only anatomic (‘type 1′) prototypes are available in our discipline. 3D-printed models gained higher acceptance among the surgical team as they provided additional haptic information and allowed surgical emulation; thus, they significantly contributed to team learning. 3D virtual modeling advances into 4D functional models in virtual/mixed reality. In combination with bioprinting and biofabrication, 3D-printed models could represent an avenue for the creation of ‘type 2′ individual cardiac implants.

## Figures and Tables

**Table 1:** Two types of 3D-printed objects in healthcare [3].

Types and Description	Examples
3D-printed anatomical prototypes of an individual patient: replicate exact patient morphology; do not come into direct contact with the patient	Anatomic models for demonstration, surgical planning, and emulations
3D-printed patient-specific medical hardware: newly-designed objects created by computer-aided design (CAD) based on and added to individual patient characteristics; direct patient contact	Customized/personalized implants Prostheses External fixators Splints Surgical instrumentation and surgical cutting aides

**Table 2:** Characteristics of congenital heart patients undergoing surgery using 3D-printed models.

No	Age (Month)	Diagnoses; Indication for a 3D-Printed Model (Bold)	Previous Surgery	3D-Printed Models		New Diagnosis	Model Assistance in	Operation Performed
				Blood Volume	Hollow			
1	6.5	HLHS; **aortic arch obstruction**	Norwood-1	Yes	Yes		Cannulation for EC circulation: method and location	Aortic arch redo; univentricular staging: BDG
2	7	HLHS; **aortic arch obstruction**	Norwood-1	Yes	No		Clarification of the geometry of obstruction	Aortic arch redo; univentricular staging: BDG
3	60	Tricuspid atresia, malposed great arteries, left PA hypoplasia; **subaortic obstruction**	Right MBTS	Yes	Yes	Origin of left mainstem coronary artery from the ascending aorta	Site of aortic opening; clarifying the location of the resection	Subaortic resection; PA plasty; univentricular staging: BDG
4	96	Tricuspid atresia, malposed great arteries; persistent pulmonary hypertension; **distal aortic arch obstruction**	Atrial septectomy, PAB DKS, BDG Take-down BDG to central MBTS	Yes	No	Kinking of the distal transverse aortic arch (v aortic coarctation)	Surgical approach (sternotomy vs. thoracotomy), cannulation site and arch repair	Distal transverse aortic arch repair; univentricular palliation: upsize of the central MBTS
5	9	Tetralogy of Fallot, hypoplastic pulmonary annulus; **uncertain coronary anatomy**	Left-sided MBTS	Only virtual 3D model created		Single left coronary artery: RCA from LAD	Need for RV-PA conduit	Biventricular complete repair with RV-PA conduit; PA-plasty
6	16	Tetralogy of Fallot with absent pulmonary valve syndrome; **obstruction of the left mainstem coronary artery**	Fallot-repair; implantation of biological pulmonary prosthesis	Only virtual 3D model created		Cause and location of left coronary artery obstruction	Cause and location of left coronary artery obstruction	Biventricular, Lecompte maneuver: placement of the dilated right PA in front of the aorta
7	19	Pulmonary atresia, VSD, MAPCAs; **complex spatial relationship of MAPCAs**	Central MBTS	Yes	No	Clarification of spatial relationship of MAPCAs	Surgical strategy of unifocalization	Biventricular staging: unifocalization, RV-PA conduit
8	15	Mesocardia, bilateral SVCs, common atrium and iAVD; **cor triatriatum sinistrum**	Atrial baffle patch; iAVD repair	Only virtual 3D model created			Anatomical landmarks for the left atrial resection	Biventricular repair: cor triatriatum repair
9	8	Dextrocardia, visceral heterotaxy, DORV/TGA; **left pulmonary branch hypoplasia**	Left-sided MBTS PDA stent	Yes	Yes	Left atrial appendage crossing the pulmonary trunk	Geometry of intracardiac pathway and pulmonary trunk augmentation	Biventricular repair: REV operation, transannular patch with monocusp; extensive PA plasty
10	13	Dextrocardia, visceral heterotaxy, right atrial isomerism, left IVC, right SVC, supracardiac TAPVD, common atrium, cAVD, DORV/TGA, pulmonary atresia; **clarification of complex segmental anatomy**	Left-sided MBTS TAPVD-repair, BDG Left PA stenting	Yes	Yes		Surgical strategy (emulation); intracardiac pathways: size and shape of patches	Biventricular repair: TAPVD unroofing to left atrium, atrial separation patch, cAVD repair-REV, BDG takedown, RV-PA conduit
11	36	Dextrocardia, venous anomalies, common atrium; **residual ASD**	Atrial baffle implantation (Mustard)	No	Yes		Surgical approach (from the left side); size/shape of the atrial patch	Biventricular repair: complete atrial baffling
12	11	Dextrocardia, visceral heterotaxy, venous anomalies, common atrium, cAVD; **uncertain segmental anatomy**	None	No	Yes	Muscular VSD	Size/shape of the atrial patch	Biventricular repair: atrial baffling (Mustard), cAVD correction
13	8	Dextrocardia, visceral heterotaxy, venous anomalies, common atrium, iAVD, pulmonary stenosis, vascular ring; **uncertain segmental anatomy**	None	Yes	Yes	Muscular VSD	Size/shape of the atrial patch	Biventricular repair: atrial baffling (Mustard), iAVD repair, pulmonary valvotomy, division of vascular ring
14	36	Dextrocardia, visceral heterotaxy, bilateral SVCs, cTGA; **uncertain intraatrial anatomy**	Bilateral BDG	Yes	Yes		Geometry of intraatrial conduit	Univentricular staging: TCPC: intracardiac conduit, PA-plasty
15	82	Mesocardia, common atrium, criss-cross heart (supero-inferior ventricles), TGA, restrictive VSD; **extent of the left ventricle thrombus and suitability for biventricular circulation**	Central MBTS BDG	Yes	Yes	Inlet VSD	Left ventricle thrombus conditions, intracardiac conduit geometry; possibility of biventricular circulation	Univentricular staging: TCPC intracardiac conduit, LV thrombus removal, VSD enlargement

Abbreviations: ASD: atrial septal defect, AV: atrioventricular, BDG: bidirectional (Glenn) superior cavopulmonary anastomosis, cAVD: complete atrioventricular defect, cTGA: congenitally corrected transposition of the great arteries, DKS: Damus–Kaye–Stansel anastomosis, DORV: double outlet right ventricle, EC: extracorporeal circulation, HLHS: hypoplastic left-heart syndrome, iAVD: incomplete atrioventricular defect, IVC: inferior vena cava, MBTS: modified Blalock-Taussig shunt, PA: pulmonary artery, PAB: pulmonary artery banding, PDA: patent arterial duct, REV: “réparation d’étage ventriculaire”, crossing of the outflow pathways at ventricular level, RV: right ventricle, SVC: superior vena cava, TAPVD: total anomalous pulmonary venous drainage, TCPC: complete cavopulmonary connection, TGA: transposition of the great arteries, VSD: ventricular septal defect.

**Table 3:** Average values of opinions of the multidisciplinary team and patient relatives on 3D modeling based on a questionnaire survey. Range of values: 1 = strongly disagree, 2 = disagree, 3 = indifferent, 4 = agree, 5 = strongly agree; n/a: non-applicable.

Questions	Multidisciplinary Team (46 Replies)	Patient Relatives (28 Replies)
3D virtual models helped understand the anatomy/clinical situation	4.8	2.7
3D-printed model provided additional information	4.1 (surgeons: 5)	4.9
Accuracy	4.1	n/a
Improved communication	4.9	5
Facilitated patient safety intraoperatively	4.9	n/a
Cost/benefit adequacy	4	n/a
Undertake the extra work associated with 3D modeling/printing	4.7	n/a
Would you assume the additional cost of 3D modeling/printing	4.1	4.8

## Data Availability

Data supporting reported results can be found in the hospital database and are kept with patient confidentiality, i.e., the data are not publicly available due to patient confidentiality reasons.

## References

[1] Senge P.M. (1990). The Fifth Discipline: The Art and Practice of the Learning Organization.

[2] Hoang D., Perrault D., Stevanovic M., Ghiassi A. (2016). Surgical applications of three-dimensional printing: A review of the current literature & how to get started. Ann. Transl. Med..

[3] Kiraly L. (2018). Three-dimensional modelling and three-dimensional printing in pediatric and congenital cardiac surgery. Transl. Pediatr..

[4] Mottl-Link S., Boettger T., Krueger J.J., Rietdorf U., Schnackenburg B., Ewert P., Berger F., Nagel E., Meinzer H.-P., Juraszek A. (2005). Images in cardiovascular medicine. Cast of Complex Congenital Heart Malformation in a Living Patient. Circulation.

[5] Biglino G., Capelli C., Wray J., Schievano S., Leaver L.-K., Khambadkone S., Giardini A., Derrick G., Jones A., Taylor A.M. (2015). 3D-manufactured patient-specific models of congenital heart defects for communication in clinical practice: Feasibility and acceptability. BMJ Open.

[6] Kurup H.K., Samuel B.P., Vettukattil J.J. (2015). Hybrid 3D printing: A game-changer in personalized cardiac medicine?. Expert Rev. Cardiovasc. Ther..

[7] Schievano S., Migliavacca F., Coats L., Khambadkone S., Carminati M., Wilson N., Deanfield J.E., Bonhoeffer P., Taylor A.M. (2007). Percutaneous pulmonary valve implantation based on rapid prototyping of right ventricular outflow tract and pulmonary trunk from MR data. Radiology.

[8] Ong C.S., Loke Y.-H., Opfermann J., Olivieri L., Vricella L., Krieger A., Hibino N. (2017). Virtual Surgery for Conduit Reconstruction of the Right Ventricular Outflow Tract. World J. Pediatr. Congenit. Hear. Surg..

[9] Schmauss D., Haeberle S., Hagl CSodian R. (2015). Three-dimensional printing in cardiac surgery and interventional cardiology: A single-centre experience. Eur. J. Cardiothorac Surg..

[10] Yoo S.-J., Thabit O., Kim E.K., Ide H., Yim D., Dragulescu A., Seed M., Grosse-Wortmann L., Van Arsdell G. (2016). 3D printing in medicine of congenital heart diseases. 3D Print. Med..

[11] Shiraishi I., Yamagishi M., Hamaoka K., Fukuzawa M., Yagihara T. (2010). Simulative operation on congenital heart disease rubber-like urethane stereolithographic biomodels based on 3D datasets of multislice computed tomography. Eur. J. Cardiothorac Surg..

[12] (2016). United Arabic Emirates Population. http://ghdx.healthdata.org/geography/united-arab-emirates.

[13] Jacobs J.P., Mayer J.E., Mavroudis C., O’Brien S.M., Austin E.H., Pasquali S.K., Hill K.D., Overman D.M., St Louis J.D., Karamlou T. (2017). The Society of Thoracic Surgeons Congenital Heart Surgery Database: 2017 Update on Outcomes and Quality. Ann. Thorac. Surg..

[14] Materialise Mimics® CT Heart Tool for Heart Chamber Segmentation: Quantitative Validation. http://www.materialise.com/en/resources/medical/white-papers/materialisewhitepapermimicscthearttoolvalidationstudy.

[15] 3D Slicer. https://www.slicer.org.

[16] Cantinotti M., Valverde I., Kutty S. (2017). Three-dimensional printed models in congenital heart disease. Int. J. Cardiovasc. Imaging.

[17] Kiraly L., Tofeig M., Jha N.K., Talo H. (2016). Three-dimensional printed prototypes refine the anatomy of post-modified Norwood-1 complex aortic arch obstruction and allow presurgical simulation of the repair. Interact. Cardiovasc. Thorac. Surg..

[18] Lau I., Sun Z. (2018). Three-dimensional printing in congenital heart disease: A systematic review. J. Med Radiat. Sci..

[19] Martelli N., Serrano C., van den Brink H., Pineau JPrognon PBorget I., El Batti S. (2016). Advantages and disadvantages od 3-dimensional printing in surgery: A systematic review. Surgery.

[20] Kappanayil M., Koneti N.R., Kannan R.R., Kottayil B.P., Kumar K. (2017). Three-dimensional-printed cardiac prototypes aid surgical decision-making and preoperative planning in selected cases of complex congenital heart diseases: Early experience and proof of concept in a resource-limited environment. Ann. Pediatr. Cardiol..

[21] Chepelev L., Wake N., Ryan J., Althobaity W., Gupta A., Arribas E., Santiago L., Ballard D.H., Wang K.C., Weadock W. (2018). Radiological Society of North America (RSNA) 3D printing Special Interest Group (SIG): Guidelines for medical 3D printing and appropriateness for clinical scenarios. 3D Print. Med..

[22] Winlaw D.S., Ayer J.G. (2020). Commentary: Three-dimensional printing for preoperative planning–Beyond illustrating the obvious. JTCVS Tech..

[23] Milano E.G., Capelli C., Wray J., Biffi B., Layton S., Lee M., Caputo M., Taylor A.M., Schievano S., Biglino G. (2018). Current and future applications of 3D printing in congenital cardiology and cardiac surgery. Br. J. Radiol..

[24] Coulson J.D., Seddon M.R., Readdy W.F. (2008). Advancing safety in pediatric cardiology–approaches developed in aviation. Congenit. Cardiol. Today.

[25] Maruthappu M., Duclos A., Lipsitz S.R., Orgill D., Carty M.J. (2015). Surgical learning curves and operative efficiency: A cross-specialty observational study. BMJ Open.

[26] Szentágothai J., Szentágothai J. (1967). Science and art. Vesalius Andreas Bruxellensis: De Humani Corporis Fabrica.

[27] Rippa Bonati M. (2010). Some Traditions Regarding the Old Anatomy Theatre of Padua University. Archiving Antico dell’Università di Padua, Atti dell’Università Artista. Raccolta Minat..

[28] Ghosh S.K. (2015). Evolution of illustrations in anatomy: A study from the classical period in Europe to modern times. Anat. Sci. Educ..

[29] Dr Maude Abbott. http://www.mcgill.ca/medicalmuseum/introduction/history/physicians/abbott.

[30] Abbott M.E. (1936). Atlas of Congenital Cardiac Disease.

[31] Binder M.D., Hirokawa N., Windhorst U., Multisensory Convergence, Integration (2009). Encyclopedia of Neuroscience.

[32] Pouget A., Deneve S., Duhamel J.R. (2002). A computational perspective on the neural basis of multisensory spatial representations. Nat. Rev. Neurosci..

[33] James T.W., Humphrey G.K., Gati J.S., Servos P., Menon R.S., Goodale M.A. (2002). Haptic study of three-dimensional objects activates extrastriate visual areas. Neuropsychologia.

[34] Lunghi C., Alais D. (2013). Touch Interacts with Vision during Binocular Rivalry with a Tight Orientation Tuning. PLoS ONE.

[35] Squeri V., Sciutti A., Gori M., Masia L., Sandini G., Konczak J. (2012). Two hands, one perception: How bimanual haptic information is combined by the brain. J. Neurophysiol..

[36] Fagan T.E., Truong U.T., Jone P.-N., Bracken J., Quaife R., Abu Hazeem A.A., Salcedo E.E., Fonseca B.M. (2014). Multimodality 3-Dimensional Image Integration for Congenital Cardiac Catheterization. Methodist DeBakey Cardiovasc. J..

[37] Biaggi P., Fernandez-Golfín C., Hahn R., Corti R. (2015). Hybrid Imaging During Transcatheter Structural Heart Interventions. Curr. Cardiovasc. Imaging Rep..

[38] RealViewHearth Surgery Using 3D Hologram. https://www.youtube.com/watch?v=K2XesWsL9Uy.

[39] Mitsuno D., Ueda K., Hirota Y., Ogino M. (2019). Effective Application of Mixed Reality Device HoloLens: Simple Manual Alignment of Surgical Field and Holograms. Plast. Reconstr. Surg..

[40] Baker C.J., Sinha R., Sullivan M.E. (2012). Development of a cardiac surgery simulation curriculum: From needs assessment results to practical implementation. J. Thorac. Cardiovasc. Surg..

[41] Oktay O., Rueckert D., Bai W., Guerrero R., Rajchl M., de Marvao A., O’Regan D., Cook S.A., Heinrich M.P., Glocker B. (2016). Stratified Decision Forests for Accurate Anatomical Landmark Localization in Cardiac Images. IEEE Trans. Med Imaging.

[42] Sciutti A., Damonte F., Alloisio M., Sandini G. (2019). Visuo-Haptic Exploration for Multimodal Memory. Front. Integr. Neurosci..

[43] Jiang T., Yu D., Wang Y., Zan T., Wang S., Li Q. (2020). HoloLens-Based Vascular Localization System: Precision Evaluation Study with a Three-Dimensional Printed Model. J. Med Internet Res..

[44] Brun H., Bugge R., Suther L., Birkeland S., Kumar R., Pelanis E. (2019). Mixed reality holograms for heart surgery planning: First user experience in congenital heart disease. Eur. Heart J. Cardiovasc. Imaging.

[45] Moro C., Phelps C., Jones D., Stromberga Z. (2020). Using Holograms to Enhance Learning in Health Sciences and Medicine. Med Sci. Educ..

[46] Lau I., Gupta A., Sun Z. (2021). Clinical Value of Virtual Reality versus 3D Printing in Congenital Heart Disease. Biomolecules.

[47] Lee S., Squelch A., Sun Z. (2021). Quantitative Assessment of 3D-printed Model Accuracy in Delineating Congenital Heart Disease. Biomolecules.

[48] Preece D., Williams S.B., Lam R., Weller R. (2013). “Let’s get physical”: Advantages of a physical model over 3D computer models and textbooks in learning imaging anatomy. Anat. Sci. Educ..

[49] Hegarty M. (2010). Components of spatial intelligence. Psychol. Learn. Motiv..

[50] Ericsson K.A. (2014). Why expert performance is special and cannot be extrapolated from studies of performance in the general population: A response to criticisms. Intelligence.

[51] Kornkasem S., Black J.B. (2015). Formation of spatial thinking skills through different training methods. Cogn. Process..

[52] ElBardissi A.W., Wiegmann D.A., Dearani J.A., Daly R.C., Sundt T.M. (2007). Application of the Human Factors Analysis and Classification System Methodology to the Cardiovascular Surgery Operating Room. Ann. Thorac. Surg..

[53] Giants of Cardiothoracic Surgery: An Interview With William Brawn. https://www.youtube.com/watch?v=eMgVoL748uI.

[54] Gardner A.K., Scott D.J., AbdelFattah K.R. (2017). Do great teams think alike? An examination of team mental models and their impact on team performance. Surgery.

[55] Tarola C.L., Hirji S., Yule S.J., Gabany J.M., Zenati A., Dias R.D., Zenati M.A. Cognitive Support to Promote Shared Mental Models during Safety-Critical Situations in Cardiac Surgery (Late Breaking Report). Proceedings of the 2018 IEEE Conference on Cognitive and Computational Aspects of Situation Management (CogSIMA).

[56] Yoo S.J., Hussein N., Peel B., Coles J., van Arsdell G.S., Honjo O., Haller C., Lam C.Z., Seed M., Barron D. (2021). 3D Modeling and Printing in Congenital Heart Surgery: Entering the Stage of Maturation. Front. Pediatr..

[57] CPT-Coding System. https://www.aapc.com/resources/medical-coding/cpt.aspx.

[58] Young R. (2018). Medical 3D Printing Is Outpacing Ways to Pay for It. Radiology Business.

[59] Cohen A., Laviv A., Berman P., Nashef R., Abu-Tair J. (2009). Mandibular reconstruction using stereolithographic 3-dimensional printing modeling technology. Oral Surg. Oral Med. Oral Pathol. Oral Radiol. Endod..

[60] Tomita K., Yano K., Hata Y., Nishibayashi A., Hosokawa K. (2015). DIEP Flap Breast Reconstruction Using 3-dimensional Surface Imaging and a Printed Mold. Plast. Reconstr. Surg. Glob. Open.

[61] Meglioli M., Naveau A., Macaluso G.M., Catros S. (2020). 3D-printed bone models in oral and cranio-maxillofacial surgery: A systematic review. 3D Print. Med..

[62] Jacobs M.L., Jacobs J.P., Thibault D., Hill K.D., Anderson B.R., Eghtesady P., Karamlou T., Kumar S.R., Mayer J.E., Mery C.M. (2021). Updating an Empirically Based Tool for Analyzing Congenital Heart Surgery Mortality. World J. Pediatr. Congen. Heart Surg..

[63] Jonas R.A., Jonas R.A. (2014). Choosing the right biomaterial. Comprehensive Surgical Management of Congenital Heart Disease.

[64] Izadifar M., Chapman D., Babyn P., Chen X., Kelly M.E. (2018). UV-Assisted 3D Bioprinting of Nanoreinforced Hybrid Cardiac Patch for Myocardial Tissue Engineering. Tissue Eng. Part C Methods.

[65] Jana S., Lerman A. (2015). Bioprinting a cardiac valve. Biotechnol. Adv..

[66] Duan B., Hockaday L.A., Kang K.H., Butcher J.T. (2012). 3D Bioprinting of heterogeneous aortic valve conduits with alginate/gelatin hydrogels. J. Biomed. Mater. Res. Part A.

[67] Ong C.S., Fukunishi T., Zhang H., Huang C.Y., Nashed A., Blazeski A., DiSilvestre D., Vricella L., Conte J., Tung L. (2017). Biomaterial-Free Three-Dimensional Bioprinting of Cardiac Tissue using Human Induced Pluripotent Stem Cell Derived Cardiomyocytes. Sci. Rep..

[68] Galantowicz M. Encouraging Six-Month Results with Gore Novel Biosynthetic Tissue Valve. https://www.prnewswire.com/news-releases/w-l-gore--associates-announces-encouraging-six-month-results-with-its-novelbiosynthetic-tissue-valve-301180050.html.

[69] Kiraly L., Vijayavenkataraman S. (2021). Biofabrication in Congenital Cardiac Surgery: A Plea from the Operating Theatre, Promise from Science. Micromachines.

[70] Wang Z., Lee S.J., Cheng H.J., Yoo J.J., Atala A. (2018). 3D bioprinted functional and contractile cardiac tissue constructs. Acta Biomater..

[71] Cai E.Z., Gao Y., Ngiam K.Y., Lim T.C. (2021). Mixed Reality Intraoperative Navigation in Craniomaxillofacial Surgery. Plast. Reconstr. Surg..

